# Collagen Q and anti-MuSK autoantibody competitively suppress agrin/LRP4/MuSK signaling

**DOI:** 10.1038/srep13928

**Published:** 2015-09-10

**Authors:** Kenji Otsuka, Mikako Ito, Bisei Ohkawara, Akio Masuda, Yu Kawakami, Ko Sahashi, Hiroshi Nishida, Naoki Mabuchi, Akemi Takano, Andrew G. Engel, Kinji Ohno

**Affiliations:** 1 Division of Neurogenetics, Center for Neurological Diseases and Cancer, Nagoya University Graduate School of Medicine, Nagoya, Japan; 2 Department of Neurology, Aichi Medical University, Aichi, Japan; 3 Department of Neurology, Gifu Prefectural General Medical Center, Gifu, Japan; 4 Department of Neurology, Okazaki City Hospital, Okazaki, Japan; 5 Department of Neurology, Meitetsu Hospital, Nagoya, Japan; 6 Department of Neurology, Mayo Clinic, Rochester, Minnesota

## Abstract

MuSK antibody-positive myasthenia gravis (MuSK-MG) accounts for 5 to 15% of autoimmune MG. MuSK and LRP4 are coreceptors for agrin in the signaling pathway that causes clustering of acetylcholine receptor (AChR). MuSK also anchors the acetylcholinesterase (AChE)/collagen Q (ColQ) complex to the synaptic basal lamina. We previously reported that anti-MuSK antibodies (MuSK-IgG) block binding of ColQ to MuSK and cause partial endplate AChE deficiency in mice. We here analyzed the physiological significance of binding of ColQ to MuSK and block of this binding by MuSK-IgG. *In vitro* plate-binding assay showed that MuSK-IgG blocked MuSK-LRP4 interaction in the presence of agrin. Passive transfer of MuSK-IgG to *Colq*-knockout mice attenuated AChR clustering, indicating that lack of ColQ is not the key event causing defective clustering of AChR in MuSK-MG. In three MuSK-MG patients, the MuSK antibodies recognized the first and fourth immunoglobulin-like domains (Ig1 and Ig4) of MuSK. In two other MuSK-MG patients, they recognized only the Ig4 domain. LRP4 and ColQ also bound to the Ig1 and Ig4 domains of MuSK. Unexpectedly, the AChE/ColQ complex blocked MuSK-LRP4 interaction and suppressed agrin/LRP4/MuSK signaling. Quantitative analysis showed that MuSK-IgG suppressed agrin/LRP4/MuSK signaling to a greater extent than ColQ.

A low-density lipoprotein receptor-related protein 4 (LRP4) forms a homodimer, which combines with a homodimer of the muscle-specific receptor tyrosine kinase (MuSK) to constitute a tetrameric protein complex on the postsynaptic membrane of the neuromuscular junction (NMJ). Agrin released from the nerve terminal of spinal motor neurons binds to LRP4, and phosphorylates MuSK^1^,^2^. Activated MuSK in concert with Dok-7 and other intracellular proteins stimulates rapsyn to concentrate and anchor acetylcholine receptor (AChR) at the postsynaptic membrane^3^. Wnt ligands also directly bind to and phosphorylate MuSK to induce AChR clustering especially at an early stage of development^4^. MuSK also binds to a small leucine-rich proteoglycan, biglycan^5^, but the functional significance of biglycan on the postsynaptic membrane is not known.

At the NMJ, three tetramers of acetylcholinesterase (AChE) are linked to the triple helical collagen Q (ColQ)^6^,^7^. AChE/ColQ complex is anchored to the synaptic basal lamina by two mechanisms. First, a pair of heparan sulfate proteoglycan-binding domains (HSPBDs) in the collagen domain of ColQ bind to heparan sulfate proteoglycans including perlecan^8^,^9^,^10^. Second, the C-terminal domain (CTD) of ColQ binds to MuSK^11^. Specific anchoring of the AChE/ColQ complex to the synaptic basal lamina requires both HSPBDs and the CTD of ColQ^10^. We and others have reported that mutations affecting the CTD compromise anchoring of AChE/ColQ complex to the NMJ^10^,^11^,^12^. In *Colq*−/− mice, membrane-bound MuSK is reduced in myotubes^13^, which likely accounts for the attenuated clustering of AChR in *Colq*−/− mice^14^.

MuSK thus binds to LRP4, Wnt ligands, biglycan, and ColQ. Binding domains of MuSK for Wnt ligands and biglycan, but not for LRP4 and ColQ, have been previously reported. The ectodomain of MuSK has three immunoglobulin (Ig)-like domains (Ig1, Ig2, and Ig3) and a frizzled-like cysteine-rich domain (Fz-CRD)^15^,^16^,^17^. Fz-CRD is composed of the C6 box carrying six cysteines and the fourth Ig-like domain (Ig4) containing four cysteines^18^,^19^. Frizzled proteins are receptors for Wnt-ligands and have ten highly conserved cysteine residues forming five disulfide bonds which are essential for forming a compact folding structure^20^. The crystal structure of MuSK Fz-CRD also indicates presence of five disulfide bonds in Fz-CRD^17^. Deletion of Fz-CRD of MuSK in mice indeed causes a drastic deficit in formation of AChR clusters^21^. We recently reported that RNA-binding proteins, hnRNP C, YB-1, and hnRNP L coordinately enhance skipping of human *MUSK* exon 10 encoding C6 box to generate a Wnt-insensitive MuSK isoform^22^. The C6-deficient MuSK isoform is unique to humans and is not present in mouse, but its functional significance remains elusive. A missense mutation I96A, but not L83A, in the Ig1 domain of MuSK prevents it from binding to LRP4 and attenuates agrin-stimulated MuSK phosphorylation^23^. The LRP4-binding domain(s) of MuSK, however, have not been thoroughly investigated. In contrast, MuSK-binding domains of LRP4 have been identified as 4th and 5th LDLa repeats close to the N-terminal end, as well as the third β-propeller domain, of LRP4^23^. We also reported that mutations in the third β-propeller domain of LRP4 in patients with congenital myasthenic syndrome compromise binding of LRP4 to MuSK^24^,^25^.

Five to 15% of patients with myasthenia gravis (MG) carry antibodies directed against MuSK^26^,^27^,^28^. MuSK-MG patients respond favorably to immunotherapy, but usually do not respond to, or are even worsened by, cholinesterase inhibitors^29^,^30^,^31^,^32^. Although anti-AChR antibodies belong to the IgG1 and IgG3 subclasses that activate complement, anti-MuSK antibodies (MuSK-IgG) largely belong to the IgG4 subclass that do not activate complement^33^,^34^. In contrast to AChR-MG, antibody-dependent complement-mediated destruction of the junctional folds is not observed in MuSK-MG patients^35^ or MuSK-IgG-injected model mice^36^. Furthermore, passive transfer to immunodeficient NOD/SCID mice of MuSK-IgG4, but not of MuSK-IgG1-3, causes MG, which provides direct evidence that MuSK-IgG acts as a blocking antibody^37^. We previously reported that MuSK-IgG blocks MuSK-ColQ interaction by an *in vitro* binding assay^38^. MuSK-IgG also blocks MuSK-LRP4 interaction in the presence of agrin by an *in vitro* binding assay^39^. Similarly, IgG4 fraction and its Fab fragments, but not IgG1-3 fractions, of MuSK-IgG block MuSK-LRP4 interaction and reduce agrin-induced AChR clustering^40^. These *in vitro* observations are corroborated in model mice^36^,^38^,^41^. Passive transfer of MuSK-IgG into C57BL/6J mice causes AChE deficiency and, to a lesser extent, AChR deficiency at the NMJ^38^. Similarlly, active immunization of complement-deficient mice with MuSK^36^, and passive transfer of MuSK-IgG to C57BL/6J mice^41^, cause loss of AChR and AChE at the NMJ. The passive transfer^38^,^41^ and active immunization^36^ models show reduced MuSK expression at the NMJ. Interestingly, bivalent MuSK-IgG produced by MuSK-immunized rabbits activates phosphorylation of MuSK but also induces downregulation of Dok-7 and internalization of MuSK^42^. However, MuSK-IgG-induced internalization of MuSK may^43^ or may not^39^,^40^ take place in model mice^43^ or model cells^39^,^40^. In contrast, monovalent MuSK-IgG directly inhibits MuSK phosphorylation^42^. As lack of ColQ in *Colq*−/− mice leads to reduced membrane-bound MuSK in myotubes^13^, reduced AChR clustering in the passive transfer and active immunization models of MuSK-MG can be attributed to blocking of either MuSK-ColQ or MuSK-LRP4 interaction. The effects of MuSK-IgG on these two interactions, however, have not been investigated.

We here demonstrate that LPR4 and ColQ bind to the Ig1 and Ig4 domains of MuSK. These domains were also recognized by MuSK-IgG in three of five MuSK-MG patients. We also asked whether passive transfer of MuSK-IgG to *Colq*−/− mice reduces AChR clustering to prove blocking of either MuSK-ColQ or MuSK-LRP4 interaction causes AChR deficiency in MuSK-MG. We found that blocking of MuSK-LPR4 interaction caused reduced AChR clustering, whereas blocking MuSK-ColQ interaction had no essential effect on AChR clustering. Although ColQ increases membrane-bound MuSK in *Colq*−/− myotubes^13^, we unexpectedly found that the CTD of ColQ blocked MuSK-LRP4 interaction by an *in vitro* plate-binding assay and suppressed agrin/LRP4/MuSK signaling in cultured cells. Quantitative comparison of purified MuSK-IgG and purified recombinant CTD of ColQ showed that MuSK-IgG blocked agrin/LRP4/MuSK signaling more than ColQ.

## Results

### MuSK-IgG blocks binding of LRP4 to MuSK in the presence of agrin

**Figure 1:**
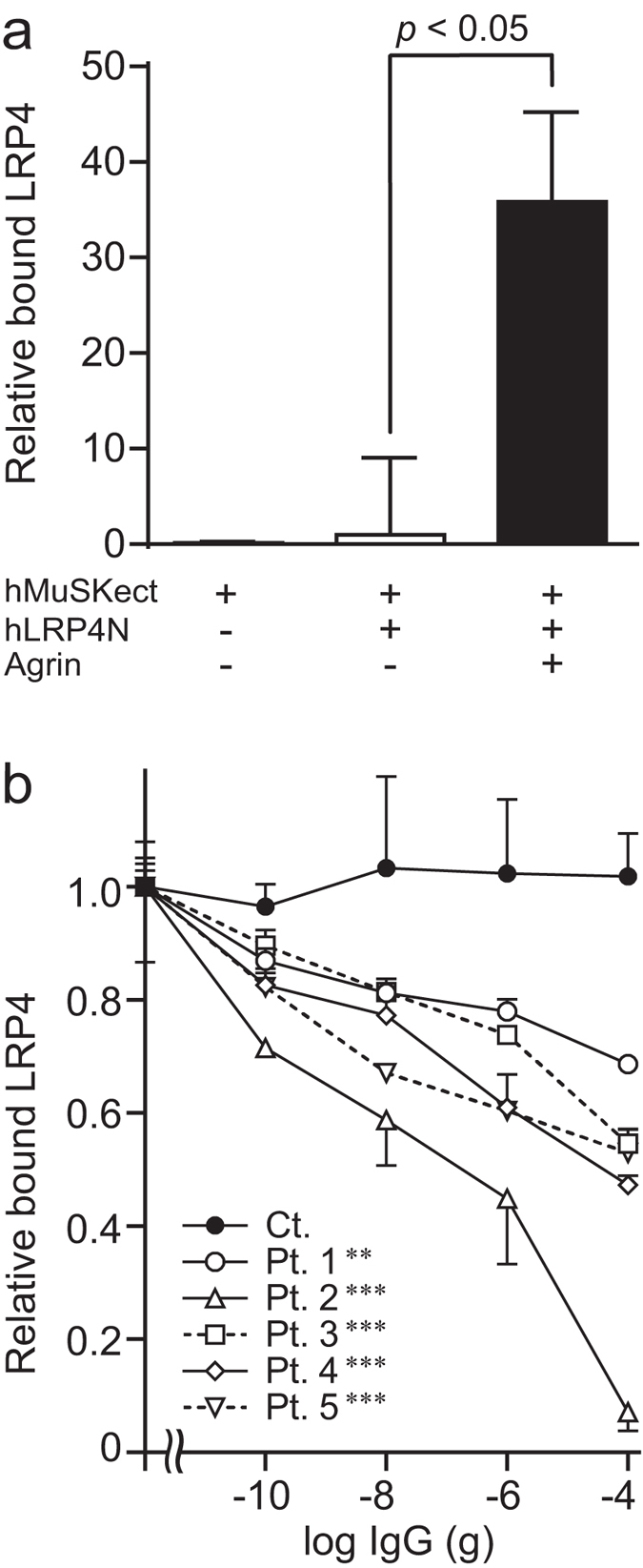
*In vitro* plate-binding assay for estimating the effect of MuSK-IgG on MuSK-LRP4 interaction in the presence of agrin. (**a**) Rat neural agrin increases the amount of purified recombinant human LRP4N-FLAG bound to the purified recombinant ectodomain of human MuSK (hMuSKect-myc) coated on a 96-well plate. Bound hLRP4N-FLAG is quantified with anti-FLAG-HRP. HRP activities are normalized for that without agrin. Mean and SEM (*n* = 3) are indicated. Statistical analysis is performed with Student’s *t*-test. (**b**) Increasing amounts of MuSK-IgG block binding of hLRP4N-FLAG to hMuSKect-myc in the presence of agrin. The amount of added IgG in a 100-μl reaction mixture is indicated in abscissa. 1 nM of IgG = 1.5 × 10^−8^ g of IgG in 100 μl. HRP activities are normalized for that at no IgG. Mean and SEM (*n* = 3) are plotted. **p < 0.01, ***p < 0.001 compared to control (Ct.) by two-way repeated measures ANOVA. Pts., patients.

Using an *in vitro* plate-binding assay, we previously reported that MuSK-IgG does not block binding of LRP4 to MuSK^38^. We now found that agrin enhanced MuSK-LRP4 interaction 36-fold (Fig. 1a). Therefore we examined whether MuSK-IgG blocks binding of LRP4 to MuSK in the presence of agrin in an *in vitro* plate-binding assay. We overlaid variable concentrations of control IgG or MuSK-IgG, as well as a fixed amount of the purified hLRP4N-FLAG, on an hMuSKect-myc-coated 96-well plate. MuSK-IgG of Patients (Pts.) 1 to 5 blocked binding of hLRP4N-FLAG to hMuSKect-myc in a dose-dependent manner, whereas control IgG did not block binding of hLRP4N-FLAG to hMuSKect-myc even at 100 μg (Fig. 1b). The degrees of inhibition of binding were variable among the five MuSK-IgGs. MuSK-IgG of Pt. 2 showed the most marked inhibition. This may represent that Pt. 2 had severe myasthenic symptoms and the residual of the plasmapheresis fluid was used for the assay. In contrast, the other Pts. were well controlled by prednisolone or in remission at the time of blood sampling.

### Passive transfer of MuSK-IgG to *Colq*−/− mice reduces AChR and MuSK expression at the NMJ, indicating that hindering MuSK-LRP4 interaction by MuSK-IgG causes reduced AChR clustering

We previously reported that passive transfer of MuSK-IgG to wild-type C57B6/J mice reduces the expression of the AChE/ColQ complex at the NMJ, and to a lesser extent the expression of AChR and MuSK at the NMJ^38^. Subsequently, reduced expression of AChE and AChR was also reported at the NMJ in model mice actively immunized with MuSK^36^ or passively immunized with MuSK-IgG^41^. The reduced expression of AChR and MuSK at the NMJ could be explained by two possible mechanisms. First, MuSK-IgG could directly hinder MuSK-LRP4 interaction, as shown above in the *in vitro* plate-binding assay. Second, MuSK-IgG could displace ColQ from MuSK, which would reduce membrane-bound MuSK and compromise AChR clustering^13^. If the first mechanism was operational in our passive transfer model of wild-type mice, passive transfer of MuSK-IgG to *Colq*−/− mice should reduce AChR and MuSK at the NMJ. In contrast, if the second mechanism was operational, passive transfer of MuSK-IgG to *Colq*−/− mice should have no effect on AChR or MuSK expression at the NMJ.

**Figure 2:**
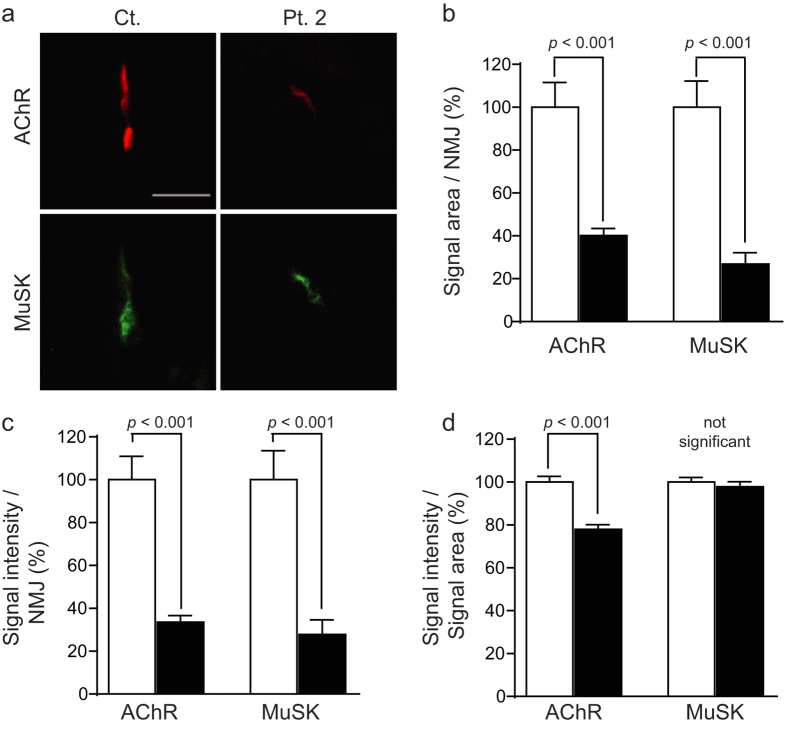
Passive transfer of IgG of control (Ct.) and patient (Pt.) 2 to *Colq*−/− mice. (**a**) Representative quadriceps muscle sections of mice injected with IgG of Ct. and Pt. 2 are stained for AChR by Alexa594-labeled α-bungarotoxin and MuSK by immunostaining. Scale bar = 40 μm. Signal areas per NMJ (**b**), intensities per NMJ (**c**), and densities (intensity/area) (**d**) of AChR and MuSK are shown by mean and SEM. We analyzed 48 NMJs (Ct.) and 42 NMJs (Pt. 2) obtained from three model mice each. Signal areas and intensities are automatically quantified with MetaMorph (Molecular Devices). Open and closed bars represent Ct. and Pt. 2, respectively. Statistical analysis is performed with Student’s *t*-test. Comparison of signal areas, intensities, and densities between wild-type model mice^38^ and *Colq*−/− model mice shown here is indicated in Supplementary Table 1.

To address this question, we examined the effect of MuSK-IgG on *Colq*−/− mice. Female *Colq*−/− mice were intraperitoneally injected with IgG isolated from a control subject (Ct-IgG) and with MuSK-IgG isolated from Pt. 2 everyday for 15 days. Expressions of AChR and MuSK were examined in the quadriceps muscle. Quantitative analysis of fluorescence signals (Fig. 2a) revealed that MuSK-IgG reduced the signal area (Fig. 2b), the signal intensity (Fig. 2c), and the signal density (signal intensity/signal area) (Fig. 2d) of AChR. In contrast, MuSK-IgG reduced the signal area (Fig. 2b) and intensity (Fig. 2c), but not the signal density (Fig. 2d), of MuSK. We previously made a MuSK-MG model mice by injecting MuSK-IgG isolated from the same Pt. 2 to wild-type C57BL/6J mice^38^. We thus compared the signal areas, intensities, and densities of AChR and MuSK between the two mouse models. We found that the signal densities of MuSK were reduced in the wild-type model mice but not in the *Colq*−/− model mice (Supplementary Table 1). ColQ physiologically increases the expression of membrane-bound MuSK^13^. In the wild-type model mice, however, MuSK-IgG displaced ColQ and the effect of ColQ on enhancing the expression of membrane-bound MuSK was lost. In contrast, in the *Colq*−/− model mice, ColQ was absent in both Ct-IgG-injected and MuSK-IgG-injected model mice, and MuSK-IgG exerted no effect on expression of ColQ. Thus, the signal density of MuSK was not changed by MuSK-IgG in the *Colq*−/− model mice. Although the significance of the difference was only slightly less than *p* = 0.05, the reduction of the AChR signal density was greater in the *Colq*−/− model mice than in the wild-type model mice (Supplementary Table 1). MuSK-IgG might have blocked MuSK-LPR4 interaction more in *Colq*−/− mice than in wild-type mice, but the exact mechanism whereby the lack of ColQ worsens the blocking effect remains unclear. To summarize, MuSK-IgG reduced the expression of MuSK and AChR at the NMJ even in *Colq*−/− mice, which supports the model, in which MuSK-IgG directly hinders MuSK-LRP4 interaction.

### MuSK-IgG binds to the Ig1 and Ig4 domains of MuSK

**Figure 3:**
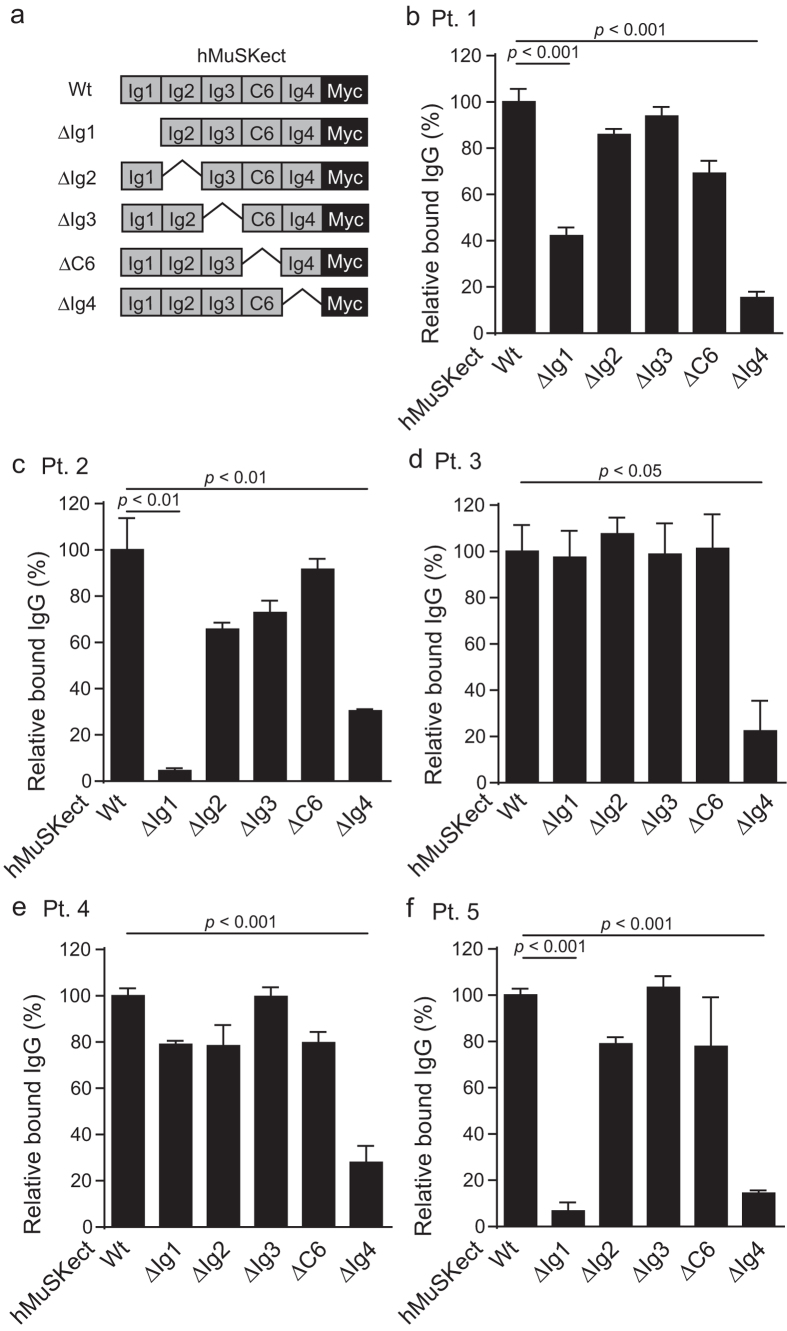
*In vitro* plate-binding assay for identification of epitopes of MuSK-IgG. Relative amounts of MuSK-IgG of Pts. 1 to 5 (**b**–*f*) bound to wild-type (Wt) and domain-deleted hMuSKect-myc (ΔIg1 to ΔIg4, and ΔC6) (a). The bound MuSK-IgG is quantified with HRP-conjugated anti-human IgG. HRP activities are normalized for that of wild-type hMuSKect-myc in each sample. Mean and SEM (*n* = 3) are plotted. Statistical significance between wild-type hMuSKect-myc and each domain-deleted hMuSKect-myc is examined with Student’s *t*-test. Only significant difference is indicated with a *p*-value.

We next examined which domain(s) of MuSK are recognized by MuSK-IgG by an *in vitro* plate-binding assay. We synthesized and purified wild-type and domain-deleted hMuSKect-myc (Fig. 3a). We then coated hMuSKect-myc on a 96-well plate, and overlaid purified total IgG of Pts. 1 to 5. In three MuSK-MG patients (Pts. 1, 2, and 5), MuSK-IgG recognized hMuSKect-myc lacking immunoglobulin-like domains 1 (ΔIg1) and 4 (ΔIg4) less efficiently than wild-type hMuSKect-myc (Fig. 3b,c,f). In two MuSK-MG patients (Pts. 3 and 4), the recognition of hMuSKect-myc by MuSK-IgG was decreased only with ΔIg4 (Fig. 3d,e). Thus, the epitopes of three MuSK-IgGs were the Ig1 and Ig4 domains of MuSK, whereas the epitope of two MuSK-IgGs was Ig4.

### ColQ and LRP4 bind to Ig1 and Ig4 domains of MuSK

**Figure 4:**
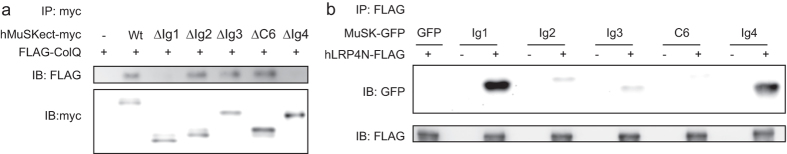
Co-immunoprecipitation of ColQ with MuSK and MuSK with LRP4. (**a**) FLAG-ColQ is immunoprecipitated (IP) with wild-type (Wt) or domain-deleted hMuSKect-myc (ΔIg1 to ΔIg4, and ΔC6). (**b**) A single MuSK domain fused to GFP (MuSK-GFP) is immunoprecipitated with hLRP4N-FLAG. MuSK-GFP has either GFP alone (GFP); or a single immunoglobulin-like domain (Ig1 to Ig4) or C6 box (C6) fused to GFP IB, immunoblotting.

We next examined which domain(s) of MuSK are recognized by ColQ and LRP4 by a co-immunoprecipitation assay. We introduced wild-type and domain-deleted ph*MUSK*ect-myc with pFLAG-*COLQ* into HEK293 cells. hMuSKect-myc was precipitated with anti-myc-antibody and co-immunoprecipitated FLAG-ColQ was examined with anti-FLAG antibody (Fig. 4a). FLAG-ColQ was able to bind to hMuSKect-myc lacking Ig2 (ΔIg2), Ig3 (ΔIg3), and C6 box (ΔC6), but not to hMuSKect-myc lacking Ig1 (ΔIg1) and Ig4 (ΔIg4).

We next introduced plasmids expressing a single MuSK domain fused to GFP along with ph*LRP4*N-FLAG into HEK293 cells. hLRP4N-FLAG was precipitated with anti-FLAG-antibody and the co-immunoprecipitated MuSK domains were examined by anti-GFP antibody (Fig. 4b). hLRP4N-FLAG was able to bind to Ig1 and Ig4, but not to Ig2, Ig3, or the C6 box. Taken together, both ColQ and LRP4 also bound to Ig1 and Ig4 of MuSK.

### ColQ blocks binding of LRP4 and MuSK

**Figure 5:**
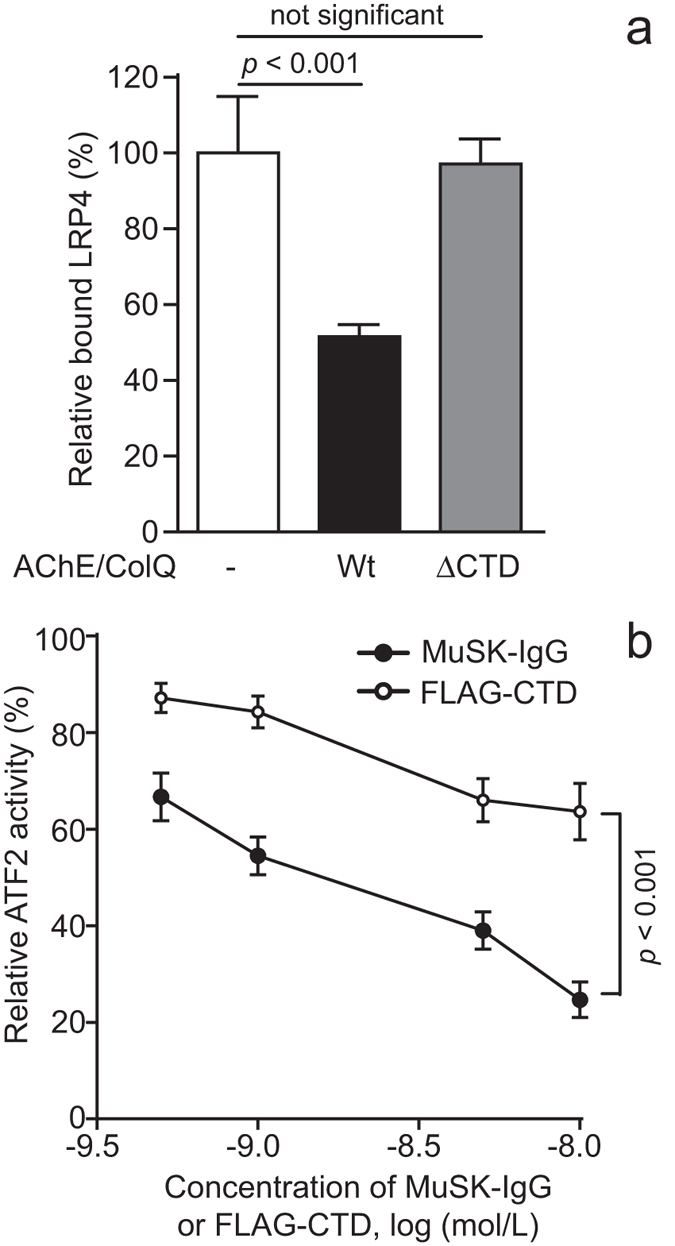
MuSK-IgG suppresses agrin/LRP4/MuSK signaling more than CTD of ColQ does. (**a**) CTD of ColQ suppresses binding of hMuSKect-myc and hLRP4N-FLAG. hMuSKect-myc is coated on a 96-well plate and hLRP4N-FLAG is overlaid in the presence of purified recombinant AChE/ColQ complex (wild-type, Wt) or purified recombinant AChE/ColQ complex lacking CTD (ΔCTD). Bound hLRP4N-FLAG is quantified with anti-FLAG-HRP. HRP activities are normalized for that without AChE/ColQ complex. Mean and SEM (*n* = 3) are indicated. Statistical analysis is performed with Student’s *t*-test. (**b**) Purified specific MuSK-IgG and purified recombinant FLAG-CTD decrease ATF2-luciferase activity, representing agrin/LRP4/MuSK signaling activity, in a dose-dependent manner in COS7 cells that are transfected with MuSK and LRP4. ATF2 luciferase activities are normalized for the Renilla luciferase activity, and also for that without MuSK-IgG or FLAG-CTD. Mean and SEM (*n* = 3) are plotted. Statistical difference is calculated by two-way repeated measures ANOVA.

We next examined the effect of the AChE/ColQ complex on LRP4-MuSK interaction by an *in vitro* plate-binding assay. We synthesized and purified wild-type hMuSKect-myc, wild-type hLRP4N-FLAG, wild-type AChE/ColQ complex, and mutant AChE/ColQ_ΔCTD complex lacking the CTD of ColQ. hMuSKect-myc was coated on a 96-well plate. AChE/ColQ complex or AChE/ColQ_ΔCTD complex was added, and subsequently purified hLRP4N-FLAG was overlaid with agrin. We found that AChE/ColQ complex hindered binding of hMuSKect-myc and hLRP4N-FLAG (Fig. 5a). In contrast, AChE/ColQ_ΔCTD complex failed to block the binding. Thus, the CTD of ColQ blocked binding of LRP4 and MuSK.

### MuSK-IgG suppresses agrin/LRP4/MuSK signaling more efficiently than the CTD of ColQ

We next quantitatively estimated the effects of MuSK-IgG and FLAG-CTD on agrin/LRP4/MuSK signaling by an ATF2-based luciferase reporter (ATF2-Luc) assay^24^. Specific MuSK-IgG of Pt. 2 was purified using MuSKect-myc-conjugated beads (Supplementary Fig. S2e). Wild-type full-length human LRP4 and wild-type full-length human MuSK were expressed along with pATF2-Luc in COS7 cells. We used COS7 cells, because COS7 cells do not express ColQ^12^,^44^. Variable concentrations of specific MuSK-IgG or purified recombinant FLAG-CTD were added to the medium along with agrin. Both specific MuSK-IgG and FLAG-CTD suppressed ATF2-Luc activity in dose-dependent manners (Fig. 5b). At 10^−8^ mol/L, MuSK-IgG suppressed ATF2-Luc activity to 24.6 ± 12.7% (mean and SD), whereas FLAG-CTD suppressed it to 63.6 ± 20.2% (mean and SD). Taken together, MuSK-IgG suppressed agrin/LRP4/MuSK signaling twice as much than the CTD of ColQ.

## Discussion

We previously reported that MuSK-IgG did not block the binding of LRP4 to MuSK *in vitro*^38^ but agrin enhanced binding of MuSK to LRP4 ~36-fold (Fig. 1a), suggesting that agrin, LRP4, and MuSK form a tertiary complex. In the presence of agrin, MuSK-IgG blocked binding of MuSK and LRP4 (Fig. 1b). Blocking by MuSK-IgG of interaction between MuSK and LRP4 in the presence of agrin has been reported by another group^39^. In a previous study in which we passively transferred MuSK-IgG to wild-type mice, we observed marked reduction of AChE/ColQ complex as well as moderate reduction of AChR and MuSK at the NMJ^38^. In *Colq*−/− mice, lack of ColQ leads to reduced AChR clustering in skeletal muscle and myotubes^13^, which is likely due to reduced membrane bound MuSK^14^. Because our previous^38^ and current *in vitro* plate-binding assays demonstrate that MuSK-IgG blocks binding of ColQ and LRP4 to MuSK, we asked whether reduced AChR clustering in our passive transfer model is either due to blocking of ColQ or LRP4. We thus made a passive transfer model with *Colq*−/− mice, and found that blocking MuSK-LRP4 interaction with MuSK-IgG is the primary cause of reduced AChR clustering (Fig. 2). Thus, MuSK-IgG reduces expression of AChE/ColQ at the NMJ, which, however, has no or minimal effect on reduced AChR clustering.

Identification of MuSK domains bound by MuSK-IgG revealed that in three patients both the Ig1 and Ig4 domains were bound by MuSK-IgG and that in two other patients only the Ig4 domain was recognized by MuSK-IgG (Fig. 3). Domains recognized by MuSK-IgG have been analyzed in three previous studies. In one study, the combined Ig1-Ig2 domains competitively blocked binding of MuSK-IgG to full-length MuSK in nine MuSK-MG patients, and Ig3-C6-Ig4 additionally showed similar competition in five of the nine patients^33^. In the second study, the Ig1-Ig2 domains were stained by sera of 33 MuSK-MG patients, and the C6-Ig4 domain was additionally stained by antibodies in 10 of the 33 patients^45^. In the third study, the ELISA assays showed that the Ig1 domain was recognized by sera of 25 MuSK-MG patients, and the Ig2 domain was additionally recognized in 5 of the 25 patients^39^. To summarize, previous reports show that the MuSK-IgG always recognizes the Ig1 domain, and in some patients it also recognizes Ig3-C6-Ig4^33^, C6-Ig4^45^, and Ig2^39^ domains of MuSK. Lack of recognition of Ig1 domain in two of our five patients is thus exceptional. The differences in the epitope specificities of the MuSK-IgG in different reports may indicate heterogeneity of the anti-MuSK antibodies. Alternatively, it may be due to the difference in the recombinant proteins used for the assay, in the assay system, in racial background, and/or in severity and duration of the disease.

We also demonstrated that the Ig1 and Ig4 domains, which were recognized by MuSK-IgG in three patients, were key domains for binding to ColQ and LRP4 (Fig. 4). Binding domains of MuSK for ColQ and LRP4 have not been thoroughly dissected. Involvement of the Ig1 domain in MuSK-LRP4 interaction was inferred from an observation that an I93A missense mutation in the Ig1 domain almost nullified MuSK-LRP4 interaction^23^. In addition to Wnt ligands^4^, LRP4^1^,^2^ and ColQ^11^, biglycan also binds to MuSK and biglycan-binding domains of MuSK have been identified^5^. Interestingly, deletion of the Ig1 domain, as well as deletion of Fz-CRD comprised of the C6 box and the Ig4 domain of MuSK, abolish binding of biglycan. Accordingly, both the Ig1 and Ig4 domains of MuSK can be shared by ColQ, LRP4, biglycan, and MuSK-IgG. Blocking MuSK-ColQ interaction that we previously reported^38^ and blocking of MuSK-LRP4 interaction that we are currently reporting are thus likely to arise from the shared binding domains. The blocking effect of MuSK-IgG on biglycan has not been studied to date. Lack of biglycan, however, displays no muscular or neuromuscular phenotypes in mice, although the growth rate and the bone mass of the mice^46^ and the expression of utrophin^47^ are reduced. The clinical significance of the effects of MuSK-IgG on biglycan, if any, is likely to be marginal. A blocking effect of MuSK-IgG on ColQ has been demonstrated in passive transfer model mice^37^,^38^,^41^ and active immunization model mice^36^. These mice show a reduced expression of AChE at the NMJ. The decay time constants of EPP are indeed prolonged in these mice^36^,^37^. MuSK-MG patients, however, do not show reduced staining for AChE in intercostal muscles^38^,^48^. Microelectrode studies of MuSK-MG patients similarly show normal decay time constants of EPP^49^ and MEPP^38^. A β2-adrenergic agonist, albuterol^50^,^51^, and another adrenergic agonist, ephedrine^52^, are effective for endplate AChE deficiency in humans, although the underlying mechanisms remain unknown. Albuterol is similarly effective in model mice passively transferred with MuSK-IgG^53^. The inefficacy of cholinesterase inhibitors in MuSK-MG patients^29^,^30^,^31^,^32^ and the beneficial effect of albuterol in model mice^53^ could be inferred from reduced AChE expression in model mice^36^,^37^,^38^,^41^. However, there is no evidence that AChE is reduced at the NMJ in MuSK-MG patients^38^,^48^. Similarly, no AChR deficiency is reported in biopsied intercostal muscle^48^ or biceps brachii muscle^35^ in MuSK-MG patients. The contradiction between patients and model mice may represent differences in species and/or analyzed muscles. MuSK expression is high in the soleus, medium in the intercostal muscle, and low in the omohyoid muscle^24^,^54^, which likely accounts for the marked bulbar and neck muscle weakness in MuSK-MG. Reduced AChE and AChR in model mice, but not in MuSK-MG patients, may be associated with different MuSK expressions in analyzed muscles.

The Ig1 domain is after the signal peptide at the N-terminal end of MuSK, whereas the Ig4 domain is at the C-terminal end of the MuSK ectodomain. As these domains flank the Ig2 and Ig3 domains, as well as the C6 box, of MuSK, ColQ, LRP4, and biglycan may require two separate binding domains. The three MuSK-MG patients may have two different species of MuSK-IgG, one for the Ig1 domain and the other for the Ig4 domain. Alternatively, the ectodomain of MuSK may form a loop so that the Ig1 domain comes close to the Ig4 domain. As MuSK forms a dimer in the postsynaptic membrane, the Ig1 domain of a MuSK may come close to the Ig4 domain of the other. If either of these is true, ColQ, LRP4, biglycan, and MuSK-IgG may recognize a combined domain comprised of Ig1 and Ig4. Crystal structures of Ig1-Ig2 domains^55^ and of Fz-CRD^17^ are solved. Crystallization of the whole ectodomain of MuSK may elucidate why two distinct domains of Ig1 and Ig4 play pivotal roles in binding to ColQ, LRP4, biglycan, and MuSK-IgG.

*In vitro* plate-binding assays revealed that the CTD of ColQ hindered MuSK-LRP4 interaction (Fig. 5a). CTD indeed suppressed agrin/LRP4/MuSK signaling (Fig. 5b). This was totally unexpected because Sigoillot and colleagues reported that membrane-bound MuSK and AChR clustering are reduced in skeletal muscle and myotubes of *Colq*−/− mice^13^. Furthermore, addition of ColQ to *Colq*-deficient myotubes partially rescues agrin-mediated AChR clustering. Their observations point to the notion that ColQ serves as an enhancer for agrin-mediated AChR clustering. ColQ thus has two opposing effects on AChR clustering: (i) upregulation of membrane-bound MuSK and enhancement of AChR clustering^13^ and (ii) blocking of MuSK-LRP4 interaction and suppression of AChR clustering. In *Colq*−/− mice, lack of enhancement of AChR clustering dominates over suppression of AChR clustering, and AChR clustering is consequently reduced. In our passive transfer model mice, partial depletion of ColQ by MuSK-IgG should suppress both of the opposing effects of ColQ on AChR clustering. Similarity of the *Colq*−/− model mice with the wild-type model mice suggests that these suppressive effects play a marginal role in defective AChR clustering in these model mice. Instead, direct hindrance of MuSK-LRP4 interaction by MuSK-IgG play a key role in reduced AChR clustering in these model mice.

**Figure 6:**
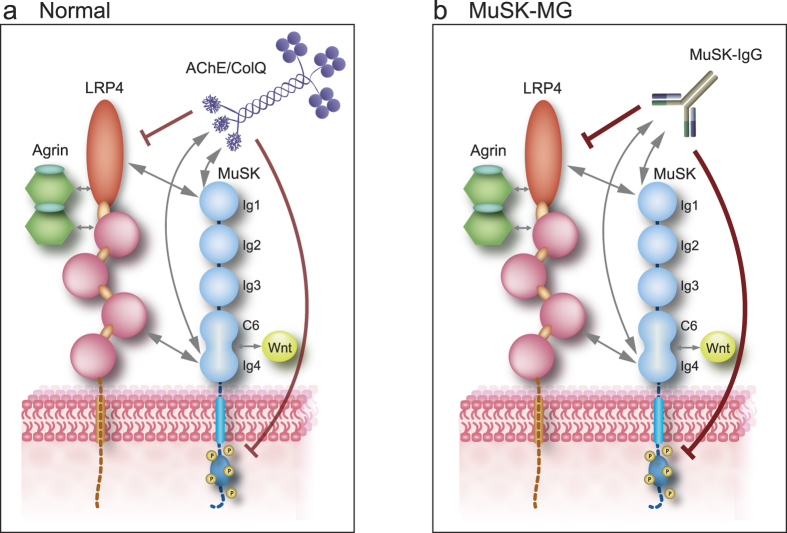
Schematics of MuSK-LRP4 interaction that is blocked by AChE/ColQ complex and MuSK-IgG. Double-headed arrows indicate putative interactions. Ig1 and Ig4 domains of MuSK bind to the 4th and 5th LDLa repeats close to the N-terminal end and the third β-propeller domain of LRP4. Mutual interactions between these domains have not been dissected. Wnt ligands bind to Fz-CRD domain of MuSK that is comprised of C6 box and Ig4^21^. Both ColQ (**a**) and MuSK-IgG (**b**) bind to Ig1 and Ig4 domains of MuSK, which blocks MuSK-LRP4 interaction and suppresses MuSK phosphorylation. MuSK-IgG (**b**) substitutes for ColQ (**a**) and directly hinders MuSK-LRP4 interaction, which exacerbates the suppression of MuSK phosphorylation. The magnitude of suppression is indicated by the thickness of the red lines.

Quantitative comparison of the suppressive effects of MuSK-IgG and ColQ on agrin/LRP4/MuSK signaling revealed that MuSK-IgG was twice as potent as ColQ in suppressing the AChR clustering signal. As MuSK-IgG is of the IgG4 subclass and acts by blocking the target epitope, ColQ is competitively displaced by MuSK-IgG. Substitution of strongly suppressive MuSK-IgG for weakly suppressive ColQ in our model mice is likely to lead to defective AChR clustering. Dissipation of ColQ-mediated enhancement of membrane-bound MuSK may also worsen defective AChR clustering, because, in contrast to ColQ, MuSK-IgG does not enhance expression of membrane-bound MuSK^39^,^40^,^43^. Taken together, substitution of MuSK-IgG for ColQ leads to enhanced suppression of MuSK signaling, as well as to lack of ColQ-mediated enhancement of membrane-bound MuSK, which are likely pathomechanisms underlying defective neuromuscular signal transmission in MuSK-MG model mice (Fig. 6). Although retained AChE and AChR in biopsied muscles in MuSK-MG patients cannot explain compromised neuromuscular signal transmission especially in bulbar muscles, heterologous overexpression of recombinant ColQ, or of recombinant CTD may partially ameliorate defective neuromuscular signal transmission caused by MuSK-IgG.

## Methods

### Standard protocol approvals and patient consents

All human studies were approved by the institutional review boards of Nagoya University Graduate School of Medicine, Aichi Medical University, Gifu Prefectural General Medical Center, Okazaki City Hospital, Meitetsu Hospital, and Mayo Clinic. Written informed consents were obtained from each patient. Animal studies were approved by the Animal Care and Use Committee of the Nagoya University. All human and animal studies were performed in accordance with the relevant governmental and institutional guidelines.

### Patients

We obtained serum from four MuSK-MG patients (Pts. 1–4) as previously reported^38^. We also obtained serum from another MuSK-MG patient (Pt. 5). We obtained 10 mL whole blood from Pts. 1, 3, 4, and 5, as well as the residual of the plasmapheresis fluid from Pt. 2. As a control (Ct.), we obtained expired fresh frozen plasma from Dr. Isao Takahashi at the Aichi Red Cross Blood Center under the institutional approval. We used sera of Pt. 2 and Ct. for all the experiments, and sera of Pts. 1, 3, 4, and 5 for the *in vitro* plate-binding assays because only small amounts of sera were available from these patients.

Ages and genders of Pts. 1 to 5 were 48F, 30F, 59M, 45F, and 40F, respectively. The titers of MuSK-IgG of Pts. 1, 2, 3, and 5 were 22.0, 11.2, 0.12, and 2.0 nM, respectively (normal < 0.01 nM). Pt. 4 was positive for MuSK-IgG, but the titer was not determined.

### Plasmids

We previously made CMV-based vectors expressing human cDNAs: pTagreT-*COLQ*^7^, pTargeT-*ACHE*^7^, pcDNA3.1-*LRP4*^24^, p3xFlag-CMV-14-*MUSK*^24^, ph*MUSK*ect-myc^38^, and ph*LRP4*N-FLAG^38^. ph*MUSK*ect-myc expresses the ectodomain of human MuSK fused to myc at the C-terminus^38^. ph*LRP4*N-FLAG expresses the N-terminal ectodomain of human LRP4 fused to FLAG at the C-terminus^38^. To generate deletion constructs (pΔIg1, pΔIg2, pΔIg3, pΔC6, and pΔIg4) from ph*MUSK*ect-myc, we deleted each domain with the QuikChange site-directed mutagenesis kit (Stratagene). The mutagenic oligonucleotides comprised of ~40 nucleotides and carried ~20-nt sequences flaking the deleted region. For generating plasmids expressing individual MuSK domains fused to GFP, the Ig1, Ig2, Ig3, C6 box, and Ig4 domains were amplified by PCR, and introduced into a mammalian expression vector pAcGFP-N1 (Takara Bio) at the NheI and XhoI sites upstream of an EGFP gene. The following constructs were generated using the QuikChange site-directed mutagenesis kit. For generating pFLAG-*COLQ*, FLAG cDNA was inserted between codons 35 and 36 in the N-terminal region of human *COLQ* in pTargeT-*COLQ*. To make pFLAG-*COLQ_*ΔCTD, we introduced the p.R315X mutation^12^ into pFLAG-*COLQ*. To generate pFLAG-CTD, the amino-terminal region and collagen domain (codons 36 to 295) were deleted from pFLAG-*COLQ*. pATF2-Luc carried an ATF2-responsive firefly luciferase cDNA that we made previously^56^. phRL-TK Renilla luciferase vector (Promega) was used as a control.

### Preparation of recombinant human AChE/ColQ complex and human AChE/ColQ_ΔCTD complex

We prepared human AChE/ColQ complex and human AChE/ColQ_ΔCTD complex for *in vitro* plate-binding assay as described previously^12^,^38^,^44^. pTargeT-*ACHE* was cotransfected with either wild-type pFLAG-*COLQ or* pFLAG-*COLQ_*Δ*CTD* into COS7 cells in a 10-cm dish using the X-tremeGene 9 transfection reagent (Roche). We extracted proteins from the cells in a buffer containing 50 mM Tris-HCl (pH 7.0), 0.5% Triton X-100, 0.2 mM EDTA, 2 μg/ml leupeptin, and 1 μg/ml pepstatin A, and 1 M NaCl. We then added four volumes of 50 mM Tris-HCl (pH 7.0) to reduce the NaCl concentration to 0.2 M. The extract was loaded onto the HiTrap Heparin HP columns (GE Healthcare). The columns were washed with five volumes of 50 mM Tris-HCl (pH 7.0) containing 0.2 M NaCl. The bound wild-type or mutant AChE/ColQ complex was eluted with 50 mM Tris-HCl (pH 7.0) containing 1 M NaCl. The eluate was concentrated with an Amicon Ultra-4 Centrifugal Filter (50 K) (Millipore) to 12-Ellman units per ml. The units were normalized with the Torpedo-derived AChE (Sigma-Aldrich).

### Preparation of wild-type and domain-deleted hMuSKect-myc protein, as well as hLRP4N-FLAG and FLAG-CTD proteins

We prepared wild-type and domain-deleted hMuSKect-myc, as well as hLRP4-FLAG and FLAG-CTD for *in vitro* plate-binding assays and the ATF2-luciferase reporter assay, as described previously^38^. Wild-type or mutant ph*MUSK*ect-myc, or ph*LRP4*N-FLAG or pFLAG-CTD, was transfected into HEK293 cells in a 10-cm dish using the calcium phosphate method^44^. We purified hMuSKect-myc with the c-myc-Tagged Protein Mild Purification Kit version 2 (MBL). We also purified hLRP4N-FLAG and FLAG-CTD with the Anti-DYKDDDDK-tag Antibody Beads (Wako). We confirmed the presence of isolated hMuSKect-myc by Western blotting with anti-myc antibody (9E10, Abcam), and hLRP4N-FLAG and FLAG-CTD with anti-FLAG antibody (M2, Sigma-Aldrich). We also confirmed the purity of isolated recombinant proteins by SDS-PAGE followed by protein staining with the Oriole Fluorescent Gel Stain (Bio-Rad).

### Purification of plasma IgG

We purified IgG as described previously^38^. We adjusted pH of plasma to 8.0 with 1 M NaOH. While stirring one volume of plasma, we slowly added 3.5 volumes of 0.4% v/v rivanol (Tokyo Chemical Industries) in 30 min. We left the solution overnight at room temperature, and removed a tenacious yellow precipitate with a sterile glass stick. After filtering the supernatant through Whatman #1 paper to remove residual precipitates, we added 8 g of activated charcoal (037–18063, Wako) for 100 ml of the IgG solution and incubated the sample overnight at 4 °C to remove rivanol. We then slowly added an equal amount of saturated ammonium sulfate, and again incubated it overnight at room temperature to precipitate the crude IgG. We centrifuged the solution at 3,000 × *g* for 30 min at room temperature, and added saline to the precipitate to form a slurry, which was then transferred to a dialysis tube (Spectrum Laboratories). The solution was dialyzed in saline at 4 °C for 3 h. The solution was then dialyzed in PBS at 4 °C for 2 h, followed by additional overnight dialysis with new PBS. We removed residual charcoals by filtering through a 0.22-μm Millex-GP filter (Millipore), and concentrated IgG using Amicon Ultra-15 Centrifugal Filter (50 K) (Millipore). We confirmed the purity of isolated IgG by 6% SDS-PAGE under a non-reducing condition followed by Coomassie staining. We also reduced IgG in 4% 2-mercaptoethanol and dissolved the heavy and light chains by 10% SDS-PAGE, which were detected by Coomassie staining.

### Purification of MuSK-specific IgG

We purified MuSK-specific IgG as described elsewhere^57^ with minor modifications. We immobilized 5-μg purified hMuSKect-myc on 1 mg of CNBr-activated Sepharose 4B (GE Healthcare) in a coupling buffer (0.1 M NaHCO_3_/Na_2_CO_3_ pH 8.3, 0.5 M NaCl) and incubated it overnight at 4 °C. Any remaining active groups on Sepharose 4B beads were blocked by incubation for 2 h at room temperature in 0.1 M Tris-HCl (pH 8.0) followed by washing with four cycles of alternating 0.1 M acetate buffer (pH 4.0) containing 0.5 M NaCl and 0.1 M Tris-HCl buffer (pH 8.0) containing 0.5 M NaCl. Purified IgG from Pt. 2 or control was added to hMuSKect-myc-immobilized sepharose beads, then incubated overnight at 4˚C with slow rotation. The beads were then washed with 0.1 M glycine-HCl buffer (pH 4.0) containing 0.5 M NaCl and 0.5% Tween-20. The bound MuSK-specific IgG was eluted with 0.1 M glycine-HCl buffer (pH 2.5). The eluate was immediately neutralized with 0.75 M Tris-HCl (pH 8.0).

### *In vitro* plate-binding assays for quantifying the effect of agrin, MuSK-IgG, and AChE/ColQ complex on LRP4-MuSK interaction

We coated the Maxi-Sorp Immuno Plate (Thermo) with 0.15 μg of purified hMuSKect-myc at 4 °C overnight, and blocked the plate with 1% BSA in PBS at room temperature for 1 h. We overlaid 0.12 μg of the purified hLRP4N-FLAG protein in each well, and incubated the mixture at room temperature for 2 h. We then quantified the bound hLRP4N-FLAG by anti-FLAG-HRP (M2, Sigma Aldrich) using the TMB substrate kit (Thermo). The HRP activities were measured with the Sunrise absorbance reader (Tecan). To examine the effect of agrin on LRP4-MuSK interaction, we added 0.24 μg rat neural agrin (550-AG-100, R&D Systems) into each well, and incubated the mixture at room temperature for 2 h. After confirming that agrin enhanced LRP4-MuSK interaction 36-fold, we added 0.24 μg rat neural agrin in each well in the following experiments. To examine the effect of MuSK-IgG on LRP4-MuSK interaction, we overlaid 1 pg to 100 μg of purified total IgG of Ct. and Pts. 1–5, and incubated the mixture for at 4˚C for 3 h. To examine the effect of AChE/ColQ complex on LRP4-MuSK interaction, we overlaid 0.12-Ellman units of AChE/ColQ complex or AChE/ColQ_ΔCTD complex, and incubated the mixture for at 4 °C for 3 h. Each time before we moved to the next step, we washed the well three times with PBS.

### *In vitro* plate-binding assay for identifying the epitopes of MuSK-IgG

We coated the Maxi-Sorp Immuno Plate (Thermo) with 3 pmol of purified wild-type or domain-deleted hMuSKect-myc at 4 °C overnight, blocked the plate with 1% BSA in PBS at room temperature for 1 h. We added 100 μg of purified total IgG of Pts. 1–5, and incubated the mixture at 4 °C for 3 h. We then quantified the bound IgG by anti-IgG4-HRP (GeneTex) using the TMB substrate kit (Thermo). The HRP activities were measured with the Sunrise absorbance reader (Tecan). Each time before we moved to the next step, we washed the plates three times with PBS.

### Passive transfer of human IgG to *Colq*−/− mice

We made passive transfer model mice as described before with minor modifications^38^. We intraperitoneally injected 35 mg IgG of Ct. and Pt. 2 into a total of six 6-week-old female *Colq*−/− mice^58^ every day for 15 days, which conformed to a guideline of pre-clinical animal model of MuSK-MG^59^. The serum IgG concentration of Pt. 2 was 4.16 mg/ml. As 35 mg IgG was given to each mouse every day, the predicted serum IgG concentration in mouse was 35 mg/13 g/0.6 = 4.49 mg/ml. Even if human IgG was not degraded in the injected mouse body, the serum IgG concentration after 15 days of IgG injection was 4.49 mg/ml x 15 = 67.4 mg/ml, which was 16 times higher than that in Pt. 2. IgG preparations were sterilized with a 0.22-μm filter (Millipore) and dissolved in 400 μl PBS. To suppress any active immune response to the human IgG, we injected 300 mg/kg of cyclophosphamide monohydrate (10 mg/ml in 0.9% NaCl) intraperitoneally 24 h after the first IgG injection^60^. The mice were sacrificed on day 16 under deep anesthesia. One of the three mice injected with MuSK-IgG showed severe paralysis of hind limbs and was sacrificed on day 8 under deep anesthesia. Skeletal muscles of mice were frozen in the liquid nitrogen-cooled isopentane and sectioned at 8-μm thick with a Leica CW3050-4 cryostat at −20 °C. Muscle section were incubated with anti-MuSK antibody (dilution 1:100, C-19, Santa Cruz) and then incubated with anti-rabbit FITC (1:100, Vector Lab.) along with α-bungarotoxin Alexa594-conjugate (Life technologies) for visualizing AChR. We quantified signals by the BX53 microscope (Olympus) equipped with imaging software MetaMorph (Molecular Devices).

### Co-immunoprecipitation to determine MuSK domains that interact with ColQ and LRP4

To determine MuSK domains that interact with ColQ, wild-type or domain-deleted ph*MUSK*ect-myc was cotransfected with pFLAG-*COLQ* in HEK293 cells in a 6-cm dish using Fugene6 Transfection Reagent (Promega). To determine MuSK domains that interact with LRP4, a plasmid harboring a single MuSK domain fused to GFP was cotransfected with ph*LRP4*N-FLAG into HEK293 cells. We extracted proteins from the cells in a buffer containing 50 mM HEPES (pH 7.0), 150 mM NaCl, 0.5% NP-40, 2 μg/ml leupeptin, and 1 μg/ml pepstatin A. We added anti-myc-antibody (9E10, Abcam) or anti-FLAG-antibody (M2, Sigma-Aldrich), and incubated the mixture at 4 °C for 2 h. We additionally added Dynabeads Protein G (Life Technologies) and incubated the mixture at 4 °C for overnight. We washed beads with a buffer containing 50 mM HEPES (pH 7.0), 150 mM NaCl 5 times, then extracted bound proteins with a buffer containing 50 mM Tris (pH 6.8), 2% SDS, 0.02% 2-mercaptoethanol at 95 °C. The precipitated and co-precipitated proteins were analyzed by Western blotting. We detected hMuSKect-myc with anti-myc antibody (A-14, Santa Cruz); FLAG-ColQ and hLRP4N-FLAG with anti-FLAG-HRP (M2, Sigma-Aldrich), and GFP-tagged MuSK single domains with anti-GFP (598, MBL).

### ATF2-based luciferase reporter assays for quantifying agrin/LRP4/MuSK signaling activity

We quantified agrin/LRP4/MuSK-mediated ATF2 activity as described previously^24^ with minor modification. We introduced pATF2-Luc, phRL-TK Renilla luciferase, pcDNA3.1-*LRP4*, p3xFlag-CMV-14-*MUSK* to COS7 cells with X-tremeGene 9 transfection reagent (Roche). At 8 h after transfection, we added 7.5 ng agrin and 0.5 nmol/L to 10 nmol/L of MuSK-specific IgG or FLAG- CTD in the medium in a 48-well plate. The cells were incubated 24 additional hours, and proteins were extracted in the passive lysis buffer (Promega). The firefly and Renilla luciferase activities were measured using the Dual luciferase system (Promega) in PowerScan Mx (DS Pharma Biomedical).

## Additional Information

**How to cite this article**: Otsuka, K. *et al.* Collagen Q and anti-MuSK autoantibody competitively suppress agrin/LRP4/MuSK signaling. *Sci. Rep.* **5**, 13928; doi: 10.1038/srep13928 (2015).

## Supplementary Material

Supplementary Information
