# Supplementary material for: Collagen Q and anti-MuSK autoantibody competitively suppress agrin/LRP4/MuSK signaling
Source: Sci Rep. 2015 Sep 10;5:13928. doi: 10.1038/srep13928 (PMC4564764; doi:10.1038/srep13928)
Supplement: Supplementary Information [file srep13928-s1.pdf]

Supplementary Information

Collagen Q and anti-MuSK autoantibody competitively suppress agrin/LRP4/MuSK signaling

Kenji Otsuka[1], Mikako Ito[1], Bisei Ohkawara[1], Akio Masuda[1], Yu Kawakami[1], Ko Sahashi[2], Hiroshi Nishida[3], Naoki Mabuchi[4], Akemi Takano[5], Andrew G. Engel[6], Kinji Ohno[1]

[1]Division of Neurogenetics, Center for Neurological Diseases and Cancer, Nagoya University Graduate School of Medicine, Nagoya, Japan
[2]Department of Neurology, Aichi Medical University, Aichi, Japan
[3]Department of Neurology, Gifu Prefectural General Medical Center, Gifu, Japan
[4]Department of Neurology, Okazaki City Hospital, Okazaki, Japan
[5]Department of Neurology, Meitetsu Hospital, Nagoya, Japan
[6]Department of Neurology, Mayo Clinic, Rochester, Minnesota

Address correspondence to Dr. Ohno, Division of Neurogenetics, Center for Neurological Diseases and Cancer, Nagoya University Graduate School of Medicine, 65 Tsurumai, Showa-ku, Nagoya 466-8550, Japan.
e-mail: ohnok@med.nagoya-u.ac.jp

Supplementary information includes:

Supplementary Figures S1-S2

Supplementary Table 1

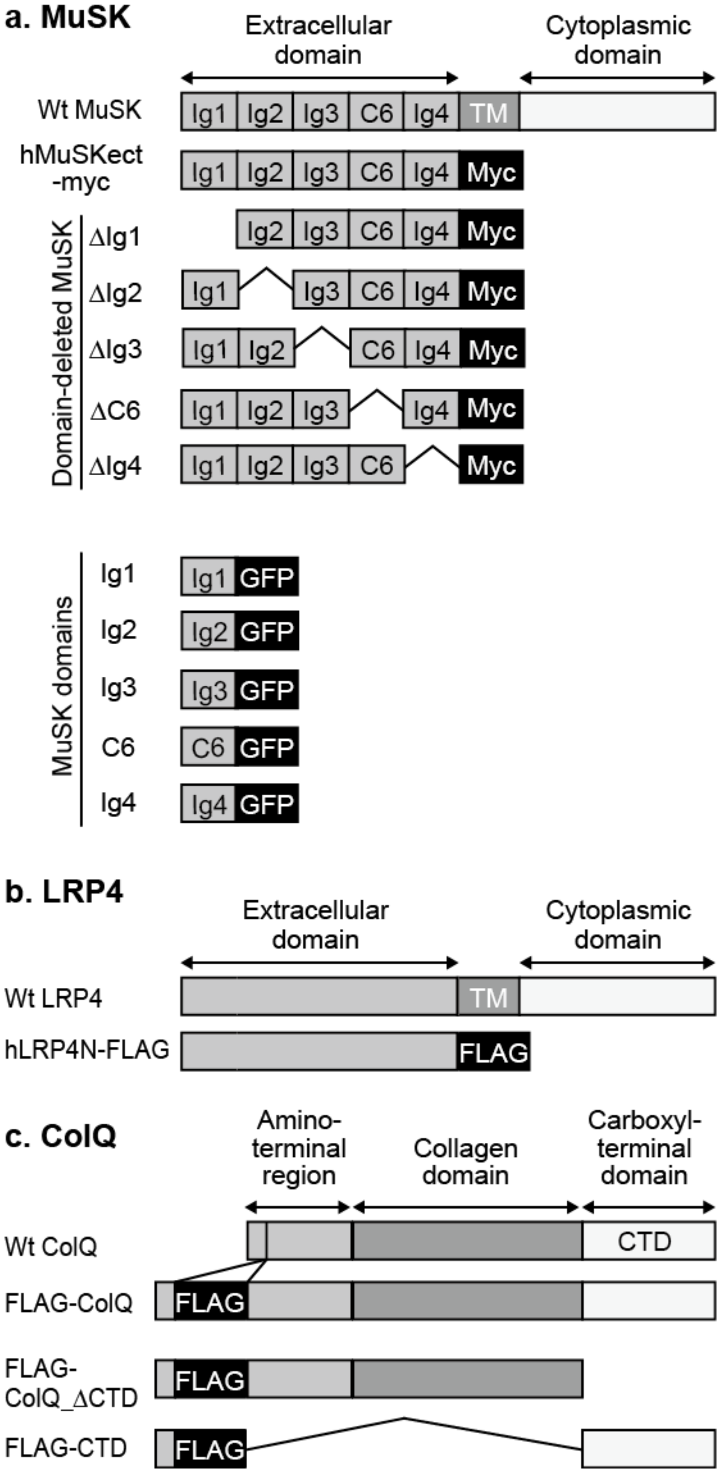

Supplementary Figure S1. Schemes of expression constructs. (a) hMuSKect-myc carries the extracellular domain of human MuSK that is fused to the myc tag. ΔIg1 to ΔIg4 lack immunoglobulin-like domains 1 to 4, respectively. ΔC6 lacks the C6 box having six cysteines. We also generated plasmids expressing individual domains (Ig1-Ig4 and C6) fused to GFP. Wt, wild-type construct. TM, transmembrane domain. (b) hLRP4N-FLAG is an N-terminal extracellular domain of LRP4 fused to FLAG. (c) FLAG is inserted between codons 105 and 106 of human ColQ to generate FLAG-ColQ. C-terminal domain (CTD) is deleted to generate FLAG-ColQ_ΔCTD. Similarly, the amino-terminal region and collagen domain is deleted to generate FLAG-CTD.

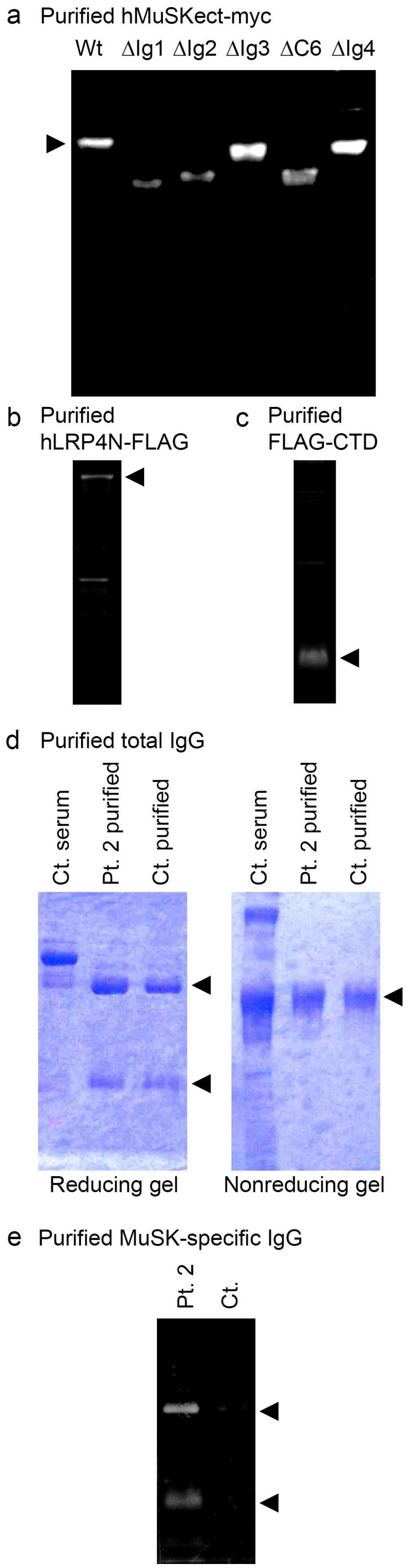

Supplementary Figure S2. The whole-protein staining of purified proteins. Reducing SDS-PAGE followed by protein staining with the Oriole Fluorescent Gel Stain (Bio-Rad) of purified wild-type and domain-deleted hMuSKect-myc (a), hLRP4-FLAG (b), FLAG-CTD (c), and MuSK-specific IgG of Pt. 2 and Ct. (e). (d) Reducing and nonreducing SDS-PAGE of control serum and purified total IgG of Pt. 2 and Ct., followed by Coomassie staining. Arrowheads point to estimated molecular weights of expected proteins. Note that domain-deleted hMuSKect-myc have lower molecular weights than wild-type hMuSKect-myc.

Supplementary Table 1. Morphometric parameters of AChR and MuSK at NMJ of wild-type and *Colq*-/- mice that are passively transferred with MuSK-IgG.

	Wild-type mice	*Colq*-/- mice	*P*
AChR signal area	31.5 ± 31.2	40.1 ± 21.2	0.152
AChR signal intensity	27.3 ± 27.6	33.5 ± 20.1	0.263
AChR signal intensity/signal area	83.9 ± 10.0	77.9 ± 13.9	0.039
MuSK signal area	37.2 ± 37.4	26.8 ± 31.9	0.210
MuSK signal intensity	30.5 ± 34.4	27.8 ± 41.1	0.761
MuSK signal intensity/signal area	80.3 ± 8.59	97.8 ± 13.6	< 0.001

For both AChR and MuSK, relative areas and intensities are compared to those in mice injected with IgG purified from Ct. serum. Mean and SD are indicated.
